# Malocclusion severity and its associations with oral health-related quality of life in an adult population

**DOI:** 10.1093/ejo/cjab070

**Published:** 2021-09-27

**Authors:** Linnea Närhi, Mimmi Tolvanen, Pertti Pirttiniemi, Anna-Sofia Silvola

**Affiliations:** Department of Oral Development and Orthodontics, Oral Health Sciences, Faculty of Medicine, University of Oulu, Medical Research Center Oulu (MRC Oulu), Oulu University Hospital, Finland; Center for Life Course Health Research, Faculty of Medicine, University of Oulu, Finland; Department of Oral Development and Orthodontics, Oral Health Sciences, Faculty of Medicine, University of Oulu, Medical Research Center Oulu (MRC Oulu), Oulu University Hospital, Finland; Department of Oral Development and Orthodontics, Oral Health Sciences, Faculty of Medicine, University of Oulu, Medical Research Center Oulu (MRC Oulu), Oulu University Hospital, Finland

## Abstract

**Aim:**

The aim of this study was to investigate malocclusion severity and its associations with oral health-related quality of life (OHRQoL) among middle-aged adults.

**Materials and methods:**

The study material consisted of 1786 subjects from the Northern Finland Birth Cohort 1966 who attended dental and oral examination as part of the 46-year-old follow-up study. Malocclusion severity was assessed using the Dental Health Component (DHC) of the Index of Orthodontic Treatment Need (IOTN) and the Peer Assessment Rating index (PAR) from digital 3D dental models. Participants also answered a questionnaire including the Oral Health Impact Profile (OHIP-14) and a question on their satisfaction with occlusal function. Differences between malocclusion severity groups were evaluated for both genders separately. For adjusted models, multivariate Poisson regression models were conducted.

**Results:**

In this study population, 31.3% had great or very great orthodontic treatment need according to DHC and the mean PAR total score was 22.05. The most severe malocclusions were associated with OHRQoL, especially the psychosocial and handicap dimensions, and satisfaction with occlusal function. There was a significant difference between genders, men having more severe malocclusion but women reporting more OHRQoL impacts.

**Conclusion:**

One third of the study population were considered to have severe malocclusion. There was an association between malocclusion severity and OHRQoL in adult population, particularly in women.

## Introduction

Unlike other oral health conditions, malocclusion is not actually a disease but rather a set of different levels of deviations from optimal occlusion. Nevertheless, the most severe malocclusions may be handicapping in the sense of the impact they have on daily life (1), and in many countries, their treatment is included in public oral health care programs. Measuring the severity of malocclusion gives an estimate of how much the case deviates from normal occlusion, and the severity of malocclusion has been considered to correlate with need for orthodontic treatment (1, 2).

The severity of malocclusion has usually been investigated using occlusal indices. Different indices have been developed for different purposes (3, 4), but most of them can be used in determining malocclusion severity and orthodontic treatment need (2, 4, 5). Using occlusal indices gives an objective professional evaluation of the level of malocclusion severity but have been criticized for lacking the subjective perspective of the individual (6, 7, 8). The professionals’ and patients’ opinion of treatment need have found to differ significantly (9). Even in severe malocclusions, treatment is not obligatory if the adult patient does not experience any harm from the malocclusion. On the other hand, minor irregularities can be of great concern for the individual, potentially affecting self-esteem and self-confidence (10, 11), and the aesthetic and psychosocial impacts of malocclusions have been found to be a common reason for seeking orthodontic treatment (12–14).

Lately, there has been increasing interest in investigating the oral health-related quality of life (OHRQoL) as it measures the individual’s own perception of oral health and physical, psychological, and social well-being (6, 7). Factors influencing the OHRQoL include caries, tooth loss, malocclusion, socioeconomic/sociodemographic factors, age, sex, culture, and expectations (15–19).

Previous reviews report evidence of the associations of malocclusions with OHRQoL (15, 20, 21). In orthodontic patients, severe malocclusion has been found to cause in particular psychosocial harm, but to have some physical impacts as well (22–24). Orthodontic/orthognathic treatment has shown to significantly improve OHRQoL (13, 25, 26), and those improvements have found to be long-term (27).

On population level, associations between malocclusion and OHRQoL have been found among children and adolescents (28–30), but there is only little evidence of these associations in adult populations (31). The findings in children may not be generalized to adult populations because the impacts of malocclusion on OHRQoL have been found to be different in different age groups (12, 15, 21), and the long-term effects of malocclusion as well as possible adaptation to the condition will only be detected over the years.

The prevalence of different levels of malocclusions is high, and the amount of the adults seeking orthodontic treatment is constantly increasing (12). Investigating the associations of malocclusion and OHRQoL in adult population provides information which can be used when developing treatment protocols and allocating resources in public oral health care.

The aim of this cross-sectional study was to investigate the severity of malocclusion and its gender-specific associations with oral health-related quality of life in a middle-aged adult population.

## Materials and methods

**Figure 1.**
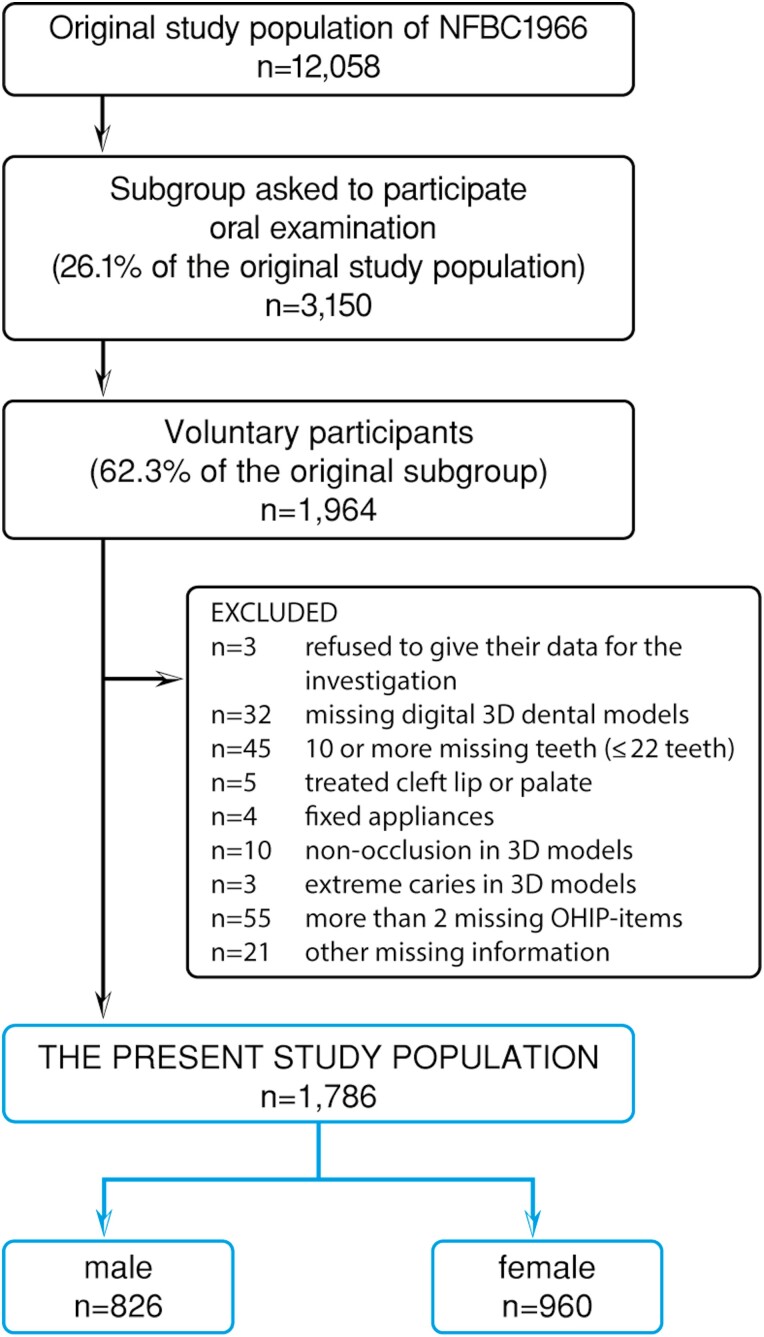
Flow chart of the study population.

This cross-sectional study was conducted as part of the Northern Finland Birth Cohort 1966 (NFBC1966) initially comprising all children from the two northern provinces of Finland (Lapland and Oulu) whose expected time of birth was in 1966 (*n* = 12 058) (32). In 2012–2013, a subgroup of 3150 subjects (consisting of all the individuals from the original study population who lived in Oulu or within 100 km from Oulu) was invited to participate in an oral examination as part of the 46-year follow-up study. Participation was voluntary, and 1964 subjects (62.3%) (912 males and 1052 females) agreed to participate. All the subjects signed a written informed consent form and had the right to refuse from participating or giving their data for the study at any time. Subjects with missing 3D dental models or other missing information, 10 or more missing teeth, cleft lip or palate, fixed appliances or non-occlusion or extreme caries were excluded from the study population. The flow chart of the study population is presented in Figure 1.

The clinical dental and oral examinations have been described in more detail previously (33, 34). Standardized self-completed questionnaires included questions concerning background information, orthodontic treatment history and OHRQoL (32, 34). Digital 3D dental models were taken using intraoral scanner (iTero 3D scanner, Cadent, San Jose, CA, USA), and the models were analysed using the 3Shape Ortho Analyzer™ software (Copenhagen, Denmark).

Severity of malocclusion was investigated using two different indices: the Dental Health Component (DHC) of the Index of Orthodontic Treatment Need (IOTN) and the Peer Assessment Rating index (PAR) (35, 36). The DHC is a five-grade index categorizing orthodontic treatment need as no need (DHC grade 1), little need (DHC grade 2), moderate need (DHC grade 3), great need (DHC grade 4), and very great need (DHC grade 5) on the basis of the worst single deviation from normal occlusion (35). In this study, DHC was trichotomized in DHC 1–2, DHC 3, and DHC 4–5 for further analyses.

The PAR index was originally developed to assess the orthodontic treatment outcome, but it has shown to be valid in assessing treatment need, as well (4). It provides a single summary score indicating all the occlusal deviations in the dentition. The PAR index consists of five components: anterior segment, buccal occlusion, overbite, overjet and centreline. All the occlusal features of these segments were scored and weighted according to British weightings, and these component scores were summed up to create a PAR total score, a higher score indicating a higher level of irregularities (36). The validity and reproducibility of both of these indices have been found to be high (35, 36). The subgroups of the best and the worst 20% extremities were separated from the PAR total scores; the cut-off points were set at PAR total score of ≤13 for the good occlusion group and PAR total score of ≥31 for the severe malocclusion group.

The PAR ratings were recorded by two examiners (L.N. and A.-S.S.) and the DHC by one examiner (L.N.) from the digital 3D dental models. Both examiners were trained and calibrated to use the indices, and interexaminer agreement was 0.901, and intraexaminer agreements varied 0.959–0.995. Digital 3D models have been found to be a valid and reliable method for assessing occlusal deviations (37, 38).

OHRQoL was measured using the Finnish translation of the 14-item Oral Health Impact Profile (OHIP-14), which has been found to be valid and reliable and to have a good precision (18, 39). The OHIP-14 consists of seven dimensions: functional limitation, physical pain, psychological discomfort, physical disability, psychological disability, social disability and handicap. Each dimension included two questions and they were answered on a five-point Likert scale (0 = never, 1 = hardly ever, 2 = occasionally, 3 = fairly often, 4 = very often) concerning the preceding month. OHIP-14 severity score is summed up from the values of the dimension scores (potential range 0–8), the potential range of OHIP-14 severity being 0–56. Higher OHIP-14 dimension and severity scores indicate worse OHRQoL (39). In addition to the OHIP-14 questions, the participants answered one separate question, ‘how satisfied are you with your occlusal function’, and the answers were coded as follows: 1 = very satisfied, 2 = quite satisfied, 3 = quite unsatisfied, and 4 = very unsatisfied.

The research program is coordinated by the Department of Health Sciences, Faculty of Medicine, University of Oulu, and the study protocol was approved by the Ethics Committee of the Northern Ostrobothnia District in Oulu, Finland (74/2011).

### Statistical analysis

The mean values of OHIP-14 severity and dimension scores, satisfaction with occlusal function scores, and PAR total and component scores were calculated. The differences in mean OHIP severity scores according to treatment need groups, education, caries and self-reported orthodontic treatment history were evaluated. The mean OHIP severity and dimension scores, satisfaction with occlusal function scores, and the mean PAR total and component scores were compared according to treatment need groups, so that the scores of moderate and great/very great treatment need groups (DHC 3, and DHC 4–5) were compared separately to the scores of the no/little treatment need group (DHC 2). Similarly, the differences in mean OHIP severity and dimension scores and satisfaction with occlusal function scores between the good occlusion group and the severe malocclusion group according to PAR total scores were evaluated. Mann–Whitney *U*-test was used to examine the differences between groups.

Poisson regression analyses were conducted to evaluate the association between malocclusion severity and OHRQoL when adjusted for confounders. The possible confounders chosen were dichotomized as follows: education (0 = polytechnic/university degree, 1 = no professional education/vocational or college-level education), presence of caries (0 = no, 1 = yes), self-reported history of orthodontics (0 = no, 1 = yes). OHIP severity and dimension scores and satisfaction with occlusal function scores were dependent variables (separate model for each) and PAR and DHC were independent variables, all as continuous count variables. Poisson regression estimates (RR) describe the RR when comparing 1-point difference in PAR or 1 grade difference in DHC. RR > 1 means that the risk for having higher OHIP scores is increased.

All statistical analyses were performed separately for both genders. *P*-values of < 0.05 were considered statistically significant. Analyses were conducted using IBM SPSS Statistics 25.0 (SPSS Inc., Chicago, IL, USA).

## Results

The characteristics of the study population are presented in Table 1. The mean OHIP severity in the study population was 3.63 (3.17 in men and 4.02 in women, *P* = 0.001). Lower OHRQoL was statistically significantly associated with caries with both genders (*P* < 0.001), and lower educational level in men (*P* = 0.018).

**Table 1.** Characteristics of the study population, and mean values of the Oral Health Impact Profile (OHIP-14) severity. Mann−Whitney *U*-test

		All			Male			Female		
		*n* (%)	OHIP severity	*P*	*n* (%)	OHIP severity	*P*	*n* (%)	OHIP severity	*P*
ALL (*n* = 1786)		1786	3.63		826	3.17	0.001*	960	4.02	0.001*
Education	Low	654 (38.9)	3.92	0.076	382 (48.5)	3.54	0.018	276 (30.6)	4.50	0.216
	Med/high	1026 (61.1)	3.37		406 (51.5)	2.73		626 (69.4)	3.78	
Caries	Yes	733 (41.3)	3.15	<0.001	394 (47.9)	3.82	<0.001	339 (35.5)	4.92	0.001
	No	1043 (58.7)	4.33		428 (52.1)	2.57		615 (64.5)	3.55	
Self-reported orthodontic treatment history	Yes	330 (18.5)	3.41	0.580	137 (16.6)	2.64	0.066	193 (20.1)	3.95	0.631
	No	1456 (81.5)	3.68		689 (83.4)	3.28		767 (79.9)	4.04	
DHC 2		461 (25.8)	3.62		175 (21.2)	3.11		286 (29.8)	3.93	
DHC 3		767 (42.9)	3.57	0.450**	384 (46.5)	3.05	0.211**	383 (39.9)	4.10	0.633**
DHC 4		512 (28.7)	3.72	0.191**	245 (29.7)	3.34	0.121**	267 (27.8)	4.07	0.406**
DHC 5		46 (2.6)	3.59	0.633**	22 (2.7)	3.91	0.958**	24 (2.5)	3.29	0.600**

*P-value for the difference between genders.

**The DHC 3, DHC 4, and DHC 5 groups compared separately to the DHC 2 group.

Of the total study population, 25.8% had no/little orthodontic treatment need (DHC 2), 42.9% had moderate treatment need (DHC 3), and 31.3% had great or very great orthodontic treatment need, considered to have a severe malocclusion (DHC 4–5). None of the subjects were scored as having DHC 1. There was a statistically significant difference in malocclusion severity between genders. Severe malocclusion was more common in men as the percentages were 21.2% for no/little need, 46.5% for moderate need, and 32.3% for great/very great need, while in women they were 29.8%, 39.9%, and 30.3%, respectively (*P* = 0.004) (Table 1).

The mean PAR total score in the study population was 22.05 (SD 10.62), and there was a statistically significant association between DHC grades and PAR total score and all PAR component scores. The mean PAR total score was higher in men (22.92, SD 10.58) compared to women (21.29, SD 10.60) (*P* = 0.001). When evaluating the malocclusion severity by DHC, severe malocclusion (DHC 4 and 5) was associated only with handicap dimension of OHIP-14 in women (*P* = 0.012), and lower satisfaction with occlusal function in both genders (*P* = 0.010 in men, *P* = 0.001 in women) (Table 2).

**Table 2.** Gender-specific mean values of the Peer Assessment Rating (PAR) total and component scores, the Oral Health Impact Profile (OHIP-14) severity and dimensions, and satisfaction with occlusal function for different orthodontic treatment need groups by Dental Health Component (DHC) of Index of Orthodontic Treatment Need (IOTN) (DHC 2 no/little need, DHC 3 moderate need, DHC 4–5 great/very great need). Mann−Whitney *U*-test

	Male					Female				
	DHC 2	DHC 3		DHC 4–5		DHC 2	DHC 3		DHC 4–5	
	*n* = 175	*n* = 384	*P*	*n* = 267	*P*	*n* = 286	*n* = 383	*P*	*n* = 291	*P*
PAR total score	13.84	21.66	<0.001*	30.70	<0.001*	12.38	20.60	<0.001*	30.96	<0.001*
Anterior segment	3.15	7.07	<0.001*	8.97	<0.001*	3.08	6.71	<0.001*	8.86	<0.001*
Buccal occlusion	3.22	3.56	0.029*	4.50	<0.001*	2.94	3.58	<0.001*	4.41	<0.001*
Overjet	4.90	7.36	<0.001*	12.74	<0.001*	4.38	7.22	<0.001*	13.57	<0.001*
Overbite	1.27	1.55	0.107	2.39	<0.001*	1.10	1.37	0.032*	2.13	<0.001*
Centreline	1.30	2.11	0.002*	2.10	0.002*	0.87	1.71	<0.001*	1.99	<0.001*
OHIP severity	3.11	3.05	0.211	3.39	0.145	3.93	4.10	0.633	4.01	0.503
Functional limitation	0.19	0.17	0.932	0.16	0.832	0.19	0.19	0.408	0.22	0.101
Physical pain	1.25	1.36	0.388	1.37	0.423	1.57	1.51	0.726	1.34	0.188
Psychological discomfort	0.66	0.66	0.509	0.76	0.171	0.93	1.00	0.693	1.04	0.317
Physical disability	0.22	0.19	0.629	0.16	0.140	0.31	0.33	0.341	0.20	0.194
Psychological disability	0.35	0.32	0.499	0.43	0.207	0.51	0.54	0.495	0.62	0.253
Social disability	0.22	0.15	0.435	0.22	0.942	0.16	0.25	0.105	0.22	0.409
Handicap	0.22	0.19	0.843	0.28	0.371	0.26	0.28	0.435	0.37	0.012*
Satisfaction with occlusal function	1.93	1.92	0.885	2.07	0.010*	1.91	2.03	0.012*	2.08	0.001*

*P*-values for the difference compared to the DHC 2 group.

*P < 0.05.

Table 3 shows the best and worst 20% extremities of PAR total scores (considered as good occlusion and severe malocclusion) and their association with OHRQoL. Women with severe malocclusion had more functional limitation, psychological discomfort and handicap (*P* = 0.021, *P* = 0.041, *P* = 0.002, respectively). In men such associations were not found. Severe malocclusion decreased satisfaction with occlusal function in both genders (*P* < 0.001). On the contrary, women with severe malocclusion reported less physical disability (*P* = 0.007).

**Table 3.** Gender-specific mean values of the Oral Health Impact Profile (OHIP-14) severity and dimensions, and satisfaction with occlusal function for the best and the worst 20% extremities of the Peer Assessment Rating (PAR) total scores: good occlusion (PAR total score ≤ 13) and severe malocclusion (PAR total score ≥ 31). Mann−Whitney *U*-test

	Male			Female		
	Good occlusion	Severe malocclusion		Good occlusion	Severe malocclusion	
	*n* = 154	*n* = 182	*P*	*n* = 233	*n* = 185	*P*
OHIP severity	3.62	3.42	0.755	3.65	4.02	0.713
Functional limitation	0.14	0.19	0.397	0.12	0.27	0.021*
Physical pain	1.49	1.32	0.239	1.60	1.29	0.070
Psychological discomfort	0.76	0.80	0.637	0.80	1.12	0.041*
Physical disability	0.26	0.14	0.081	0.37	0.18	0.007*
Psychological disability	0.43	0.47	0.359	0.46	0.60	0.229
Social disability	0.27	0.19	0.988	0.13	0.18	0.665
Handicap	0.27	0.31	0.393	0.17	0.37	0.002*
Satisfaction with occlusal function	1.90	2.12	<0.001*	1.90	2.14	<0.001*

*P*-values for the difference between malocclusion severity groups.

*P < 0.05.

**Figure 2.**
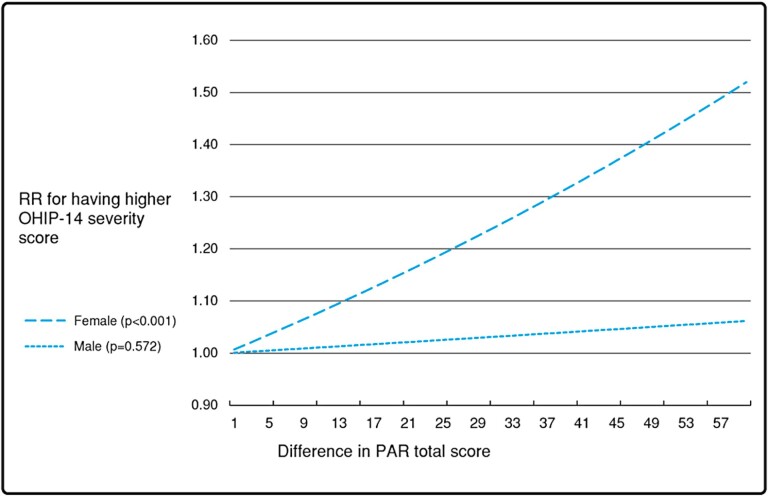
Relative risk for having higher Oral Health Impact Profile severity score according to difference in Peer Assessment Rating total score, based on the final logistic regression model, adjusted for education, caries, and self-reported orthodontic treatment history.

The associations of malocclusion severity with OHRQoL and satisfaction with occlusal function adjusted for selected confounding variables are presented in Table 4. When analysing the gender differences, women reported more effects on OHRQoL. In men, DHC was associated with OHIP severity, psychological disability and handicap (*P* = 0.002, *P* = 0.006, *P* = 0.041), and PAR only with psychological disability (*P* = 0.029). In women, DHC was associated with psychological disability and handicap (*P* = 0.042, *P* = 0.004), and PAR with OHIP severity (*P* < 0.001), functional limitation (*P* = 0.001), psychological discomfort (*P* < 0.001), psychological disability (*P* = 0.001), social disability (*P* = 0.007), handicap (*P* < 0.001), and satisfaction with occlusal function (*P* = 0.040). Negative associations (worse occlusion associated with better OHRQoL) of DHC and physical pain (*P* = 0.008) and of DHC and PAR with physical disability (*P* = 0.014, *P* = 0.022) were found in women. In men, there was a negative association of PAR with physical disability (*P* = 0.006). Figure 2 illustrates the RRs when comparing patients according to difference in their PAR total score (1–60 points difference). Especially in women, the risk for higher OHIP severity score is increased significantly when having higher PAR total scores.

**Table 4.** Gender-specific final Poisson regression models for the Oral Health Impact Profile (OHIP-14) severity and dimensions, and satisfaction with occlusal function, adjusted for education, caries and self-reported orthodontic treatment history. Independent variables: malocclusion severity defined with Peer Assessment Rating (PAR) (scores 0–60) and the Dental Health Component (DHC) (grades 2–5) indices

		Male (*n* = 784)			Female (*n* = 897)		
Dependent variable	Independent variables	RR	95% CI	P	RR	95% CI	P
OHIP severity	PAR	1.001	0.997–1.005	0.572	1.007	1.004–1.010	<0.001
	DHC	1.085	1.031–1.142	0.002	1.012	0.972–1.053	0.564
Functional limitation	PAR	1.010	0.995–1.027	0.197	1.022	1.008–1.035	0.001
	DHC	0.957	0.767–1.194	0.697	1.087	0.911–1.296	0.354
Physical pain	PAR	0.997	0.991–1.003	0.291	0.996	0.991–1.001	0.147
	DHC	1.049	0.969–1.135	0.236	0.914	0.856–0.976	0.008
Psychological discomfort	PAR	1.006	0.998–1.014	0.143	1.015	1.009–1.021	<0.001
	DHC	1.108	0.993–1.237	0.067	1.063	0.981–1.151	0.133
Physical disability	PAR	0.977	0.961–0.993	0.006	0.985	0.973–0.997	0.014
	DHC	0.901	0.727–1.118	0.345	0.836	0.718–0.974	0.022
Psychological disability	PAR	1.012	1.001–1.023	0.029	1.014	1.006–1.022	0.001
	DHC	1.235	1.062–1.436	0.006	1.115	1.004–1.239	0.042
Social disability	PAR	0.990	0.974–1.006	0.220	1.018	1.005–1.032	0.007
	DHC	1.160	0.938–1.435	0.170	1.127	0.949–1.340	0.173
Handicap	PAR	1.013	0.999–1.027	0.062	1.024	1.013–1.035	<0.001
	DHC	1.224	1.008–1.486	0.041	1.229	1.067–1.415	0.004
Satisfaction with occlusal function	PAR	1.004	0.999–1.009	0.092	1.005	1.000–1.009	0.040
	DHC	1.041	0.975–1.110	0.228	1.046	0.989–1.106	0.115

RR, relative risk; CI, confidence interval.

## Discussion

The aim of this study was to investigate the severity of malocclusion and its gender-specific associations with OHRQoL in a Finnish adult population. The prevalence of severe malocclusions was relatively high in the present study population, and the total OHIP-14 score was 3.63, corroborating the findings in a previous population-based study (31). This study confirms that especially the most severe malocclusions have a negative impact on OHRQoL. There were also significant gender differences in both malocclusion severity and its associations with OHRQoL.

Malocclusions have been found to be generally associated with OHRQoL among orthodontic patients (14, 22–24), and those associations have also been discovered on population level (17, 28, 30, 31). The present study shows that severe malocclusion is associated with OHRQoL, and with satisfaction with occlusal function in a middle-aged adult population. Previous population-based studies have found associations of malocclusion with social and emotional well-being among children and adolescents (20, 28). However, studies in adult patients and in adult populations have reported functional and physical dimensions to be affected as well (19, 22–24, 31), which is in line with the findings of this study.

Associations of malocclusion severity with OHRQoL and satisfaction with occlusal function were found in both genders, though, significant gender differences in the associations of malocclusion severity and OHRQoL were also revealed. Women with severe malocclusions reported significantly more OHRQoL impacts than men, mostly in psychological and handicap dimensions, but also in overall OHIP severity and functional limitation. The same tendency was seen in both unadjusted and adjusted models, more clearly after adjusting with confounding variables. In the regression models, higher PAR total scores were associated with a risk for having higher OHIP scores particularly in women. Females have previously been found to be more sensitive to OHRQoL impacts, especially related to aesthetics or psychosocial well-being (14, 22, 30), though the evidence of gender differences has been inconsistent (23). One third of the study population had severe malocclusion or great/very great orthodontic treatment need (DHC 4 and 5), consistent with a previous study in a representative adult population (40). However, the prevalence of severe malocclusions in previous population-based studies varies widely (29, 41–43). The occlusal indices have been found to correlate highly with each other (42), which was seen in this study as well. The mean PAR total score in the present study population was relatively high, similarly to a previous study in adult population using the PAR index (42). The severity of malocclusion differed by gender, males having slightly higher percentage of severe malocclusions (DHC 4 and 5) than females. Likewise, the mean PAR total score in males was slightly higher compared to females. Parallel findings have been observed in Italian population (44) while no gender difference in DHC was seen among German adults (40). Anyhow, the difference between genders seems to be minor, and the clinical importance is limited.

According to the PAR index, the score limit for good occlusion is considerably low, and the recommended cut-off point for orthodontic treatment need has been more than or equal to 17 (2, 4, 42). In the present study, over two thirds of the study population had a PAR total score ≥ 17, which differs significantly from a study among children and adolescents in Hong Kong, where more than half of the population had a PAR total score ≤ 10 (29). This may be related to the composition of the PAR index as it sums up all the occlusal deviations, and in a population of 46 year olds, there may be numerous smaller irregularities even in acceptable occlusions. The high prevalence of severe malocclusion in this study population may be related to the low prevalence of orthodontic treatment history (18.5%).

DHC has clear cut-off points for different treatment needs. Though DHC is a normative objective measure, the same DHC score represents a variety of conditions, and individuals with the same DHC score may in reality differ in their malocclusion severity and actual treatment need. In DHC, only the one worst occlusal deviation is recorded, which is not necessarily the one causing harm or about which the individual might be concerned (11, 45). Unlike DHC, the PAR index represents all the occlusal deviations in the dentition, and thus seems to be a better predictor of malocclusions’ impacts on OHRQoL in the adjusted models in this study.

There are several other factors affecting OHRQoL. Therefore, participants with missing teeth, cleft lip and palate and ongoing orthodontic treatment were excluded from this study (8, 16, 18, 46, 47). Previous orthodontic treatment, oral health and socioeconomic factors were taken into consideration as confounding factors in the adjusted models, as these have found to have OHRQoL impacts (8, 17, 19). The prevalence of orthodontic treatment history in this study population was considerably low, which may have some effect on the individuals’ expectations for the occlusion and accordingly to OHRQoL.

The strength of this study was the nationally representative study population, all from the same region and of the same age. The sample size was considerably large and enabled taking confounding factors into account. This is the first population-based study using the PAR index to evaluate the severity of malocclusion in such a large number of participants. In measuring the severity of malocclusion and OHRQoL, the most commonly used indices were used, and the digital 3D models enabled precise measurements (38). Both DHC and PAR have been found to be excellent indices in revealing orthodontic treatment need (2, 4, 5), and DHC has also been validated for use in epidemiological studies (3).

Although OHIP-14 is one of the most widely used OHRQoL measures, it is an instrument with functional bias, and it might be considered not a very sensitive instrument for measuring OHRQoL related to orthodontic problems (48). There are other measures available more specific for this purpose, but the Finnish translations were not available at the time of the data collection (49).

The post hoc power analyses were performed, showing good power (>80%) in the comparisons between genders and in the association between malocclusion severity and satisfaction with occlusal function. Anyhow, the power was weak (<10%) in the associations between OHIP and malocclusion severity. This confirms that even though the sample size was calculated to be sufficient, the differences in OHRQoL measured with OHIP-14 on population level are small to be detected. In this study, the mean OHIP scores were relatively low, and the differences between groups were rather small. In population-based studies, the mean OHIP scores tend to be low in general (31), and therefore, even the small differences should be taken into consideration. Anyhow, it should be noted that the associations found in this study were statistically significant albeit weak, and the clinical relevance of the differences is limited.

Thus, the associations between malocclusions measured with the indices and OHRQoL seems to be relatively weak and inconsistent. When the associations of clinically registered malocclusion traits (overjet, overbite, crossbite, scissor bite) have previously been investigated, more associations with OHRQoL and its dimensions were found (50). This confirms the significance of registering different malocclusion traits when evaluating the orthodontic treatment need and its impacts on patients’ perception. The definition of acceptable occlusion may also differ between countries, and the indices do not necessarily meet the local orthodontic treatment need criteria (51, 52).

There has been a lack of studies of malocclusions’ impacts on OHRQoL in adults, but the present study showed that there is a weak association of severe malocclusions with OHRQoL in a middle-aged adult population. Subjects with malocclusion over a long period of time tend to have higher prevalence of OHRQoL impacts (12), but in the present study population, most of the subjects do not seem to have impacts related to their occlusal deviations. Anyhow, this study corroborates the findings that the normative measures of orthodontic treatment need may not alone capture the individuals’ perspective of the condition (8).

## Conclusions

- The mean PAR total score in the study population was 22.05 (SD 10.62), and the prevalence of severe malocclusion was relatively high.
- The findings of this study suggest that there is a statistically significant albeit weak association between malocclusion severity and OHRQoL in adults on population level.
- Malocclusion severity and the associations with OHRQoL differ between genders, men having more severe malocclusion, while women experience more harm related to malocclusions.

## Data Availability

NFBC data are available from the University of Oulu, Infrastructure for Population Studies. Permission to use the data can be applied for research purposes via electronic material request portal. In the use of data, we follow the EU general data protection regulation (679/2016) and Finnish Data Protection Act. The use of personal data is based on cohort participant’s written informed consent at his/her latest follow-up study, which may cause limitations to its use. Please contact NFBC project centre (NFBCprojectcenter@oulu.fi) and visit the cohort website (www.oulu.fi/nfbc) for more information.

## References

[1] Proffit, W.R., Fields, H.W. Jr and Moray, L.J. (1998) Prevalence of malocclusion and orthodontic treatment need in the United States: estimates from the NHANES III survey. The International Journal of Adult Orthodontics and Orthognathic Surgery, 13, 97–106. 9743642

[2] Firestone, A.R., Beck, F.M., Beglin, F.M. and Vig, K.W. (2002) Evaluation of the peer assessment rating (PAR) index as an index of orthodontic treatment need. American Journal of Orthodontics and Dentofacial Orthopedics, 122, 463–469. 12439473 10.1067/mod.2002.128465

[3] Cardoso, C.F., Drummond, A.F., Lages, E.M., Pretti, H., Ferreira, E.F. and Abreu, M.H. (2011) The Dental Aesthetic Index and dental health component of the Index of Orthodontic Treatment Need as tools in epidemiological studies. International Journal of Environmental Research and Public Health, 8, 3277–3286. 21909306 10.3390/ijerph8083277 PMC3166742

[4] Khandakji, M.N. and Ghafari, J.G. (2020) Evaluation of commonly used occlusal indices in determining orthodontic treatment need. European Journal of Orthodontics, 42, 107–114. 31185083 10.1093/ejo/cjz042

[5] Beglin, F.M., Firestone, A.R., Vig, K.W., Beck, F.M., Kuthy, R.A. and Wade, D. (2002) A comparison of the reliability and validity of 3 occlusal indexes of orthodontic treatment need. American Journal of Orthodontics and Dentofacial Orthopedics, 122, 463–469. 11552122 10.1067/mod.2001.116401

[6] Allen, P.F . (2003) Assessment of oral health related quality of life. Health and Quality of Life Outcomes, 1, 40. 14514355 10.1186/1477-7525-1-40 PMC201012

[7] Sischo, L. and Broder, H.L. (2011) Oral health-related quality of life: what, why, how, and future implications. Journal of Dental Research, 90, 1264–1270. 21422477 10.1177/0022034511399918 PMC3318061

[8] de Oliveira, C.M. and Sheiham, A. (2003) The relationship between normative orthodontic treatment need and oral health-related quality of life. Community Dentistry and Oral Epidemiology, 31, 426–436. 14986910 10.1046/j.1600-0528.2003.00002.x

[9] Ghijselings, I., Brosens, V., Willems, G., Fieuws, S., Clijmans, M. and Lemiere, J. (2014) Normative and self-perceived orthodontic treatment need in 11- to 16-year-old children. European Journal of Orthodontics, 36, 179–185. 23761429 10.1093/ejo/cjt042

[10] Klages, U., Bruckner, A. and Zentner, A. (2004) Dental aesthetics, self-awareness, and oral health-related quality of life in young adults. European Journal of Orthodontics, 26, 507–514. 15536839 10.1093/ejo/26.5.507

[11] Gavric, A., Mirceta, D., Jakobovic, M., Pavlic, A., Zrinski, M.T. and Spalj, S. (2015) Craniodentofacial characteristics, dental esthetics-related quality of life and self-esteem. American Journal of Orthodontics and Dentofacial Orthopedics, 147, 711–718. 26038075 10.1016/j.ajodo.2015.01.027

[12] Neely, M.L., Miller, R., Rich, S.E., Will, L.A., Wright, W.G. and Jones, J.A. (2017) Effect of malocclusion on adults seeking orthodontic treatment. American Journal of Orthodontics and Dentofacial Orthopedics, 152, 778–787. 29173857 10.1016/j.ajodo.2017.04.023

[13] Yassir, Y.A., McIntyre, G.T. and Bearn, D.R. (2020) The impact of labial fixed appliance orthodontic treatment on patient expectation, experience, and satisfaction: an overview of systematic reviews. European Journal of Orthodontics, 42, 223–230. 31147683 10.1093/ejo/cjz043

[14] Feu, D., de Oliveira, B.H., de Oliveira Almeida, M.A., Kiyak, H.A. and Miguel, J.A. (2010) Oral health-related quality of life and orthodontic treatment seeking. American Journal of Orthodontics and Dentofacial Orthopedics, 138, 152–159. 20691356 10.1016/j.ajodo.2008.09.033

[15] Kragt, L., Dhamo, B., Wolvius, E.B. and Ongkosuwito, E.M. (2016) The impact of malocclusions on oral health-related quality of life in children-a systematic review and meta-analysis. Clinical Oral Investigations, 20, 1881–1894. 26635095 10.1007/s00784-015-1681-3 PMC5069349

[16] Steele, J.G., Sanders, A.E., Slade, G.D., Allen, P.F., Lahti, S., Nuttall, N. and Spencer, A.J. (2004) How do age and tooth loss affect oral health impacts and quality of life? A study comparing two national samples. Community Dentistry and Oral Epidemiology, 32, 107–114. 15061859 10.1111/j.0301-5661.2004.00131.x

[17] Vedovello, S.A., Ambrosano, G.M., Pereira, A.C., Valdrighi, H.C., Filho, M.V. and Meneghim, M.d.e.C. (2016) Association between malocclusion and the contextual factors of quality of life and socioeconomic status. American Journal of Orthodontics and Dentofacial Orthopedics, 150, 58–63. 27364206 10.1016/j.ajodo.2015.12.022

[18] Lahti, S., Suominen-Taipale, L. and Hausen, H. (2008) Oral health impacts among adults in Finland: competing effects of age, number of teeth, and removable dentures. European Journal of Oral Sciences, 116, 260–266. 18471245 10.1111/j.1600-0722.2008.00540.x

[19] Choi, S.H., Kim, B.I., Cha, J.Y. and Hwang, C.J. (2015) Impact of malocclusion and common oral diseases on oral health-related quality of life in young adults. American Journal of Orthodontics and Dentofacial Orthopedics, 147, 587–595. 25919104 10.1016/j.ajodo.2014.12.025

[20] Dimberg, L., Arnrup, K. and Bondemark, L. (2015) The impact of malocclusion on the quality of life among children and adolescents: a systematic review of quantitative studies. European Journal of Orthodontics, 37, 238–247. 25214504 10.1093/ejo/cju046

[21] Sun, L., Wong, H.M. and McGrath, C.P. (2017) Relationship between the severity of Malocclusion and oral health related quality of life: A systematic review and meta-analysis. Oral Health & Preventive Dentistry, 15, 503–517. 28944350 10.3290/j.ohpd.a38994

[22] Rusanen, J., Lahti, S., Tolvanen, M. and Pirttiniemi, P. (2010) Quality of life in patients with severe malocclusion before treatment. European Journal of Orthodontics, 32, 43–48. 19726489 10.1093/ejo/cjp065

[23] Hassan, A.H. and Amin, Hel-S. (2010) Association of orthodontic treatment needs and oral health related quality of life in young adults American Journal of Orthodontics and Dentofacial Orthopedics, 137, 42–47. 20122429 10.1016/j.ajodo.2008.02.024

[24] Chen, M., Feng, Z.C., Liu, X., Li, Z.M., Cai, B. and Wang, D.W. (2015) Impact of malocclusion on oral health-related quality of life in young adults. The Angle Orthodontist, 85, 986–991. 25531421 10.2319/101714-743.1 PMC8612031

[25] Andiappan, M., Gao, W., Bernabé, E., Kandala, N.B. and Donaldson, A.N. (2015) Malocclusion, orthodontic treatment, and the Oral Health Impact Profile (OHIP-14): systematic review and meta-analysis. The Angle Orthodontist, 85, 493–500. 25157973 10.2319/051414-348.1 PMC8612413

[26] de Araujo, C.M., Schroder, A.G.D., de Araujo, B.M.M., Cavalcante-Leão, B.L., Stechman-Neto, J., Zeigelboim, B.S., Santos, R.S. and Guariza-Filho, O. (2020) Impact of orthodontic-surgical treatment on quality of life: a meta-analysis. European Journal of Orthodontics, 42, 281–289. 31784741 10.1093/ejo/cjz093

[27] Paunonen, J., Svedström-Oristo, A.L., Helminen, M. and Peltomäki, T. (2020) Quality of life several years after orthodontic-surgical treatment with bilateral sagittal split osteotomy. Acta Odontologica Scandinavica, 78, 358–361. 32037937 10.1080/00016357.2020.1725110

[28] Bittencourt, J.M., Martins, L.P., Bendo, C.B., Vale, M.P. and Paiva, S.M. (2017) Negative effect of malocclusion on the emotional and social well-being of Brazilian adolescents: a population-based study. European Journal of Orthodontics, 39, 628–633. 28371848 10.1093/ejo/cjx020

[29] Sun, L., Wong, H.M. and McGrath, C.P.J. (2020) A cohort study of factors that influence oral health-related quality of life from age 12 to 18 in Hong Kong. Health and Quality of Life Outcomes, 18, 65. 32156276 10.1186/s12955-020-01317-z PMC7063806

[30] Sardenberg, F., Cavalcante-Leão, B.L., Todero, S.R., Ferreira, F.M., Rebellato, N.L. and Fraiz, F.C. (2017) A population-based study on the impact of orofacial dysfunction on oral health-related quality of life among Brazilian schoolchildren. Acta Odontologica Scandinavica, 75, 173–178. 28064555 10.1080/00016357.2016.1275038

[31] Masood, M., Suominen, A.L., Pietila, T. and Lahti, S. (2017) Malocclusion traits and oral health-related quality of life in Finnish adults. Community Dentistry and Oral Epidemiology, 45, 178–188. 28083893 10.1111/cdoe.12276

[32] University of Oulu: Northern Finland Birth Cohort 1966. University of Oulu. http://urn.fi/urn:nbn:fi:att:bc1e5408-980e-4a62-b899-43bec3755243.

[33] Alaraudanjoki, V., Laitala, M.L., Tjäderhane, L., Pesonen, P., Lussi, A. and Anttonen, V. (2016) Association of erosive tooth wear and dental caries in Northern Finland Birth Cohort 1966 – an epidemiological cross-sectional study. BMC Oral Health, 17, 6. 27430337 10.1186/s12903-016-0232-x PMC5477799

[34] Krooks, L., Pirttiniemi, P., Kanavakis, G. and Lähdesmäki, R. (2016) Prevalence of malocclusion traits and orthodontic treatment in a Finnish adult population. Acta Odontologica Scandinavica, 74, 362–367. 26940248 10.3109/00016357.2016.1151547

[35] Brook, P.H. and Shaw, W.C. (1989) The development of an index of orthodontic treatment priority. European Journal of Orthodontics, 11, 309–320. 2792220 10.1093/oxfordjournals.ejo.a035999

[36] Richmond, S., Shaw, W.C., O’Brien, K.D., Buchanan, I.B., Jones, R., Stephens, C.D., Roberts, C.T. and Andrews, M. (1992) The development of the PAR Index (Peer Assessment Rating): reliability and validity. European Journal of Orthodontics, 14, 125–139. 1582457 10.1093/ejo/14.2.125

[37] Mayers, M., Firestone, A.R., Rashid, R. and Vig, K.W. (2005) Comparison of peer assessment rating (PAR) index scores of plaster and computer-based digital models. American Journal of Orthodontics and Dentofacial Orthopedics, 128, 431–434. 16214623 10.1016/j.ajodo.2004.04.035

[38] Kiviahde, H., Bukovac, L., Jussila, P., Pesonen, P., Sipilä, K., Raustia, A. and Pirttiniemi, P. (2018) Inter-arch digital models vs. manual cast measurements: accuracy and reliability. The Journal of Craniomandibular & Sleep Practice, 36, 222–227. 10.1080/08869634.2017.1344811 28659050

[39] Slade, G.D . (1997) Derivation and validation of a short-form oral health impact profile. Community Dentistry and Oral Epidemiology, 25, 284–290. 9332805 10.1111/j.1600-0528.1997.tb00941.x

[40] Bock, J.J., Czarnota, J., Hirsch, C. and Fuhrmann, R. (2011) Orthodontic treatment need in a representative adult cohort. Journal of Orofacial Orthopedics, 72, 421–433. 22124507 10.1007/s00056-011-0047-y

[41] Kerosuo, H., Kerosuo, E., Niemi, M. and Simola, H. (2000) The need for treatment and satisfaction with dental appearance among young Finnish adults with and without a history of orthodontic treatment. Journal of Orofacial Orthopedics, 61, 330–340. 11037685 10.1007/pl00001903

[42] Soh, J., Sandham, A. and Chan, Y.H. (2005) Malocclusion severity in Asian men in relation to malocclusion type and orthodontic treatment need. American Journal of Orthodontics and Dentofacial Orthopedics, 128, 648–652. 16286213 10.1016/j.ajodo.2005.05.045

[43] Abu Alhaija, E.S., Al-Nimri, K.S. and Al-Khateeb, S.N. (2004) Orthodontic treatment need and demand in 12-14-year-old north Jordanian school children. European Journal of Orthodontics, 26, 261–263. 15222709 10.1093/ejo/26.3.261

[44] Ciuffolo, F., Manzoli, L., D’Attilio, M., Tecco, S., Muratore, F., Festa, F. and Romano, F. (2005) Prevalence and distribution by gender of occlusal characteristics in a sample of Italian secondary school students: a cross-sectional study. European Journal of Orthodontics, 27, 601–606. 16009668 10.1093/ejo/cji043

[45] Bernabé, E., de Oliveira, C.M. and Sheiham, A. (2008) Comparison of the discriminative ability of a generic and condition-specific OHRQoL measure in adolescents with and without normative need for orthodontic treatment. Health Qual Outcomes, 6, 64. 10.1186/1477-7525-6-64 PMC2533301 18718004

[46] Hunt, O., Burden, D., Hepper, P. and Johnston, C. (2005) The psychosocial effects of cleft lip and palate: a systematic review. European Journal of Orthodontics, 27, 274–285. 15947228 10.1093/ejo/cji004

[47] Liu, Z., McGrath, C. and Hägg, U. (2011) Changes in oral health-related quality of life during fixed orthodontic appliance therapy: an 18-month prospective longitudinal study. American Journal of Orthodontics and Dentofacial Orthopedics, 139, 214–219. 21300250 10.1016/j.ajodo.2009.08.029

[48] Liu, Z., McGrath, C. and Hägg, U. (2011) Associations between orthodontic treatment need and oral health-related quality of life among young adults: does it depend how you assess them? Community Dent Oral Epidemiology, 39,137–44. 10.1111/j.1600-0528.2010.00573.x 21210961

[49] Campos, L.A., Kämäräinen, M., Silvola, A.S., Marôco, J., Peltomäki, T. and Campos, J.A.D.B. (2020) Orofacial Esthetic Scale and Psychosocial Impact of Dental Aesthetics Questionnaire: development and psychometric properties of the Finnish version. Acta Odontologica Scandinavica, 28, 1–9. 10.1080/00016357.2020.1857435 33370538

[50] Silvola, A.S., Närhi, L., Tolvanen, M. and Pirttiniemi, P. (2020) Gender-specific associations of malocclusion traits with oral health-related quality of life in a Finnish adult population. European Journal of Orthodontics, 42, 242–249. 31119283 10.1093/ejo/cjz026

[51] Svedström-Oristo, A.L., Pietilä, T., Pietilä, I., Vahlberg, T., Alanen, P. and Varrela, J. (2009) Acceptability of dental appearance in a group of Finnish 16- to 25-year-olds. The Angle Orthodontist, 79, 479–483. 19413382 10.2319/040108-184.1

[52] Johansson, A.M. and Follin, M.E. (2009) Evaluation of the Dental Health Component, of the Index of Orthodontic Treatment Need, by Swedish orthodontists. European Journal of Orthodontics, 31, 184–188. 19126819 10.1093/ejo/cjn094

